# Effects of Empagliflozin in Women and Men With Heart Failure and Preserved Ejection Fraction

**DOI:** 10.1161/CIRCULATIONAHA.122.059755

**Published:** 2022-09-13

**Authors:** Javed Butler, Gerasimos Filippatos, Tariq Jamal Siddiqi, João Pedro Ferreira, Martina Brueckmann, Edimar Bocchi, Michael Böhm, Vijay K. Chopra, Nadia Giannetti, Tomoko Iwata, James L. Januzzi, Sanjay Kaul, Ileana L. Piña, Piotr Ponikowski, Ursula Rauch-Kröhnert, Sanjiv J. Shah, Michele Senni, Mikhail Sumin, Subodh Verma, Jian Zhang, Stuart J. Pocock, Faiez Zannad, Milton Packer, Stefan D. Anker

**Affiliations:** Department of Medicine, University of Mississippi School of Medicine, Jackson (J.B., T.J.S.).; Baylor Scott and White Research Institute, Dallas, TX (J.B).; National and Kapodistrian University of Athens School of Medicine, Athens University Hospital Attikon, Greece (G.F.).; Université de Lorraine, Inserm, Centre d’Investigations Cliniques, Nancy, France (J.P.F., F.Z.).; Inserm U1116, CHRU, F-CRIN INI-CRCT (Cardiovascular and Renal Clinical Trialists), Nancy, France (J.P.F., F.Z.).; Cardiovascular Research and Development Center, Department of Surgery and Physiology, Faculty of Medicine of the University of Porto, Portugal (J.P.F.).; Boehringer Ingelheim International GmbH, Ingelheim, Germany, and First Department of Medicine, Faculty of Medicine Mannheim, University of Heidelberg, Mannheim, Germany (M. Brueckmann).; Heart Failure Department, Heart Institute (InCor) do Hospital das Clínicas da Faculdade de Medicina da Universidade de São Paulo, Brazil (E.B.).; Klinik für Innere Medizin III, Universitätsklinikum des Saarlandes, Saarland University, Homburg/Saar, Germany (M. Böhm).; Max Superspeciality Hospital, Saket, New Delhi, India (V.K.C.).; Division of Cardiology, McGill University Health Centre, Montreal, QC, Canada (N.G.).; Boehringer Ingelheim Pharma GmbH & Co KG, Biberach, Germany (T.I.).; Massachusetts General Hospital and Baim Institute for Clinical Research, Boston (J.L.J.).; Cedars-Sinai Medical Center, Los Angeles, CA (S.K.).; Central Michigan University, Mount Pleasant (I.L.P.).; Wroclaw Medical University, Poland (P.P.).; Department of Cardiology, Charité University Medicine Berlin, Campus Benjamin-Franklin, Germany (U.R.-K.).; German Centre for Cardiovascular Research partner site Berlin (U.R.-K.).; Northwestern University Feinberg School of Medicine, Chicago, IL (S.J.S.).; Cardiovascular Department, Cardiology Division, Papa Giovanni XXIII Hospital, Bergamo, Italy (M. Senni).; Boehringer Ingelheim International GmbH, Ingelheim, Germany (M. Sumin).; Division of Cardiac Surgery, St. Michael’s Hospital, University of Toronto, ON, Canada (S.V.); Fuwai Hospital Chinese Academic of Medical Science, Beijing, China (J.Z.).; Department of Medical Statistics, London School of Hygiene and Tropical Medicine, UK (S.J.P.).; Baylor Heart and Vascular Institute, Baylor University Medical Center, Dallas, TX (M.P.).; Imperial College, London, UK (M.P.).; Department of Cardiology and Berlin Institute of Health Center for Regenerative Therapies, Germany (S.D.A.).; German Centre for Cardiovascular Research partner site Berlin (S.D.A.).; Charité Universitätsmedizin Berlin, Germany (S.D.A.).

**Keywords:** empagliflozin, health status, heart failure, hospitalization, men, women

## Abstract

Women and men with heart failure (HF) and preserved ejection fraction may differ in their clinical characteristics and their response to therapy. The aim of this study was to evaluate the influence of sex on the effects of empagliflozin in patients with HF and preserved ejection fraction enrolled in the EMPEROR-Preserved trial (Empagliflozin Outcome Trial in Patients With Chronic Heart Failure With Preserved Ejection Fraction).

**Methods:**

The effects of empagliflozin on the primary outcome of cardiovascular death or hospitalization for HF and on secondary outcomes (including total HF hospitalization, cardiovascular and all-cause mortality, and Kansas City Cardiomyopathy Questionnaire scores) were compared in women and men in the overall cohort and in subgroups defined by left ventricular ejection fraction (41%–49%, 50%–59%, and ≥60%). The effects of empagliflozin on physiological measures, including changes in systolic blood pressure, uric acid, hemoglobin, body weight, and natriuretic peptide levels, were also assessed.

**Results:**

Of the 5988 patients randomized, 2676 (44.7%) were women. In the placebo arm, women tended to have lower risk for adverse outcomes, including a lower risk of all-cause mortality (hazard ratio, 0.69 [95% CI, 0.56, 0.84]). Compared with placebo, empagliflozin reduced the risk of cardiovascular death or hospitalization for HF to a similar degree in both sexes (hazard ratio, 0.81 [95% CI, 0.69, 0.96] for men; and hazard ratio, 0.75 [95% CI, 0.61, 0.92] for women; *P*_interaction_=0.54). Sex did not modify the relationship between empagliflozin and outcomes across ejection fraction groups. Similar results were seen for secondary outcomes and physiological measures. Compared with placebo, empagliflozin improved the Kansas City Cardiomyopathy Questionnaire Clinical Summary Score to a similar extent in both sexes (1.38 for men versus 1.63 for women at 52 weeks; *P*_interaction_=0.77); the results were similar for Kansas City Cardiomyopathy Questionnaire overall summary score and total summary score.

**Conclusions:**

Empagliflozin produced similar benefits on outcomes and health status in women and men with HF and preserved ejection fraction.

**Registration:**

URL: https://www.clinicaltrials.gov; Unique identifier: NCT03057951.

**Clinical Perspective**

**What Is New?**

- In EMPEROR-Preserved (Empagliflozin Outcome Trial in Patients with Chronic Heart Failure with Preserved Ejection Fraction), empagliflozin reduced the risk of the primary outcome of cardiovascular death or hospitalization for heart failure to a similar degree in both women and men with heart failure and preserved ejection fraction (HFpEF), regardless of baseline left ventricular ejection fraction.
- Empagliflozin produced comparable benefits for the prespecified secondary outcomes (total heart failure hospitalization, cardiovascular death, and all-cause mortality), physiological measures, and health status in women and men with HFpEF.
- The pattern of the effects of empagliflozin in HFpEF in both sexes in EMPEROR-Preserved stands in contrast to the influence of sex on the response to neprilysin inhibition.

**What Are the Clinical Implications?**

- Because the clinical benefits of empagliflozin in patients with HFpEF are consistent in both women and men, the decision about the use of empagliflozin in patients with HFpEF should be made independently of the patient’s sex.

Patients with heart failure and preserved ejection fraction (HFpEF) are more likely to be women than men.^1,2^ Yet, despite a higher burden of comorbidities and symptoms and worse health-related quality of life, women with HFpEF have better survival and lower rates of heart failure (HF) hospitalizations than men.^3^ This may be related to differences between the sexes in the pattern of left ventricular remodeling in response to load and aging, yielding smaller left ventricular volumes and higher left ventricular ejection fractions (LVEFs) in women than men.^4–6^ Women show greater increases in left ventricular filling pressures after blood volume expansion and have greater arterial stiffness.^4–6^ In addition, women are also more predisposed to epicardial and intramyocardial fat expansion and proinflammatory imbalances in adipocyte-associated mediators.^4–7^ Given these differences, it seems plausible that the response to treatments for HFpEF differs between the 2 sexes. Indeed, in the PARAGON-HF trial (Efficacy and Safety of LCZ696 Compared to Valsartan, on Morbidity and Mortality in Heart Failure Patients With Preserved Ejection Fraction), compared with men, women experienced greater reductions in HF hospitalization but smaller improvements in health status with sacubitril/valsartan.^8^

The EMPEROR-Preserved trial (Empagliflozin Outcome Trial in Patients With Chronic Heart Failure With Preserved Ejection Fraction) studied the sodium glucose cotransporter-2 inhibitor empagliflozin in patients with HFpEF and an LVEF >40% and showed a significant reduction in the risk of cardiovascular death or HF hospitalization. We examined the influence of sex on the natural history of patients with HFpEF and on prespecified clinical outcomes, health status, and physiological biomarkers.

## Methods

### Study Design and Patient Population

The design of the EMPEROR-Preserved trial has been described previously.^9^ In brief, participants were men or women ≥18 years of age who have chronic HF with New York Heart Association (NYHA) functional class II to IV symptoms and an LVEF of >40% with no previous measurement of ≤40%. Patients were also required to have an elevated NT-proBNP (N-terminal pro-B-type natriuretic peptide) level (>900 or >300 pg/mL in patients with and without atrial fibrillation, respectively) and a documented hospitalization for HF or evidence of structural heart disease within 12 months before enrollment. Patients were randomized to receive either placebo or empagliflozin 10 mg daily for a median of 26 months. The ethics committee at each center approved the trial, and all patients provided written informed consent.

### Trial Outcomes

The outcomes for this analysis included the primary outcome, which was time to cardiovascular death or HF hospitalization, and secondary outcomes, including total HF hospitalization, cardiovascular and all-cause mortality, health status as measured by Kansas City Cardiomyopathy Questionnaire (KCCQ), and NYHA functional class. The KCCQ scores were summarized as total symptom score consisting of symptom frequency and burden; a clinical summary score consisting of physical limitation and total symptom score; and an overall summary score, which combines the clinical summary score, quality of life, and social limitation domains. Physiological measures included body weight, systolic blood pressure, NT-proBNP, uric acid, and hematocrit at 52 weeks. Change in diuretic therapy (increase in dose, decrease in dose, initiation, and permanent discontinuation) and safety outcomes, which included any adverse events, serious adverse events, volume depletion, acute renal failure, confirmed hypoglycemia, genital infection, and bone fractures, were also reported.

### Statistical Analyses

Baseline characteristics and differences between women and men were analyzed with descriptive statistics. Categorical variables were compared with the χ^2^ test, and continuous variables were compared with the *t* test. The natural history of HFpEF in the 2 sexes was assessed by studying events in the placebo arm. Time to first event outcomes was analyzed with a Cox regression model, and total (first and recurrent) hospitalizations for HF were evaluated using the joint frailty model with cardiovascular death as competing risk. Analyses were done according to the intention-to-treat principle, and hazard ratios (HRs) and 95% CIs were calculated to estimate the treatment effect of empagliflozin. Models were adjusted for age, estimated glomerular filtration rate, LVEF, region, and diabetes. The influence of sex on the effect of empagliflozin versus placebo on prespecified outcomes was studied using treatment-by-sex interaction terms for the overall population. In addition, subgroup analyses were done by LVEF subgroups (41%–49%, 50%–59%, and ≥60%) among each sex and by 6 categories of combination of sex and LVEF. The effect of empagliflozin by baseline LVEF as a continuous variable was studied for men and women separately and assessed by including the baseline LVEF-by-treatment-by-sex interaction term and interaction of these components in addition. Changes in KCCQ scores, body weight, systolic blood pressure, NT-proBNP, uric acid, and hematocrit were estimated with a mixed model for repeated measurements. NYHA functional class was analyzed with a partial proportional odds regression model adjusted for the same variables used in Cox regression model and baseline NYHA class, assuming proportionality for all covariates except region and baseline NYHA class. All analyses were conducted with SAS, version 9.4 (SAS Institute). The analyses requested by the authors were performed by the sponsor; authors had access to all analysis results.

### Data-Sharing Statement

To ensure independent interpretation of clinical study results and to enable authors to fulfill their role and obligations under the International Committee of Medical Journal Editors criteria, Boehringer Ingelheim grants all external authors access to relevant clinical study data. In adherence with the Boehringer Ingelheim Policy on Transparency and Publication of Clinical Study Data, scientific and medical researchers can request access to clinical study data after publication of the primary article in a peer-reviewed journal, regulatory activities are complete, and other criteria are met. Researchers should use the online link^10^ to request access to study data and visit the online site^11^ for further information.

## Results

### Baseline Characteristics

Overall, 2676 (44.7%) women and 3312 (55.3%) men were enrolled in the EMPEROR-Preserved trial. Baseline characteristics according to sex and to sex and LVEF category are shown in Table 1 and Table S1, respectively. Women were older and had higher body mass index and LVEF and lower KCCQ scores than men; compared with men, women were more likely to have nonischemic cause of HF, hypertension, and worse NYHA class and more likely to be treated with diuretics. Systolic blood pressure, NT-proBNP, uric acid, and hematocrit in both sexes were similar.

**Table 1.**
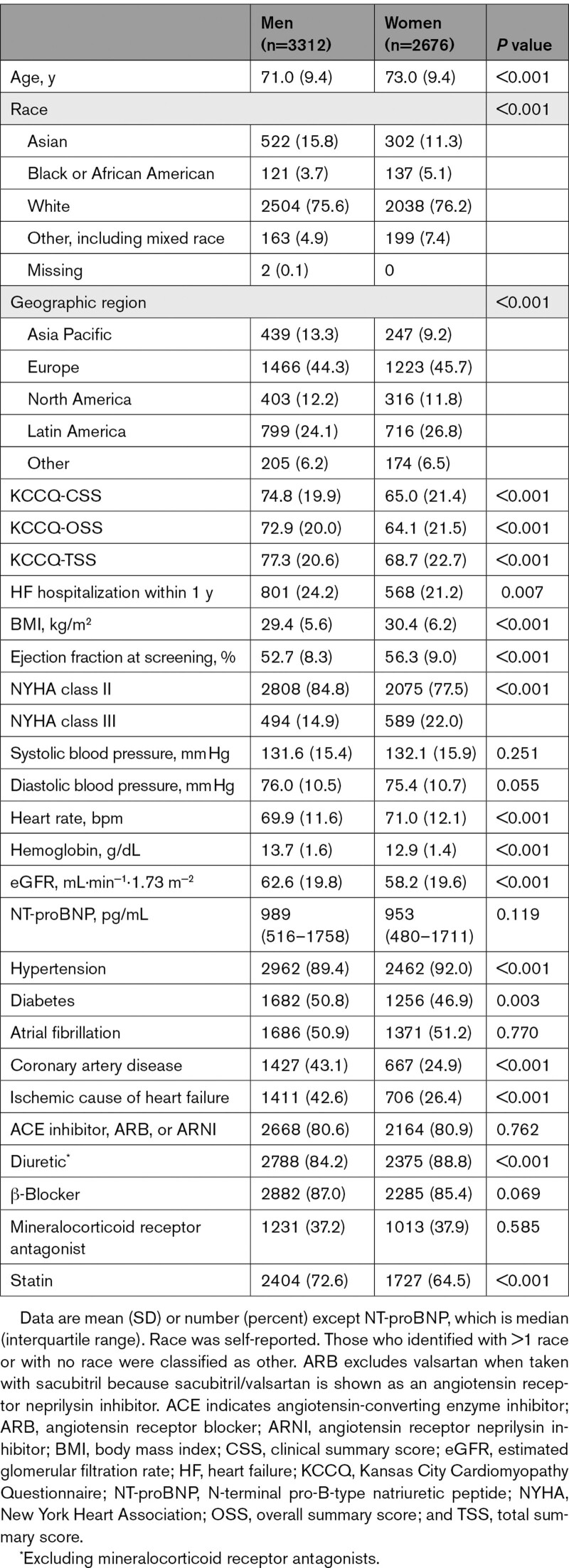
Baseline Characteristics of Patients According to Sex

### Outcomes by Sex in the Placebo Arm

When treated with placebo and compared with men, women had a numerically lower risk of cardiovascular death and hospitalizations for HF (HR, 0.87 [95% CI, 0.73, 1.05]), total hospitalizations for HF (HR, 0.83 [95% CI, 0.65, 1.07]), first HF hospitalization (HR, 0.93 [95% CI, 0.75, 1.16]), and cardiovascular death (HR, 0.79 [95% CI, 0.61, 1.03]). All-cause mortality in women was significant lower (HR, 0.69 [95% CI, 0.56, 0.84]). In the placebo group, KCCQ clinical summary score improved less in women than in men at 52 weeks (mean difference, −0.83 [95% CI, −2.11, −0.46]). Results were consistent for KCCQ overall summary score and total symptom score and at 12 and 32 weeks. Changes in body weight, systolic blood pressure, uric acid, hematocrit, and NT-proBNP during follow-up were similar in both sexes.

### Influence of Sex on the Effect of Empagliflozin

#### Cardiovascular Outcomes

**Figure 1.**
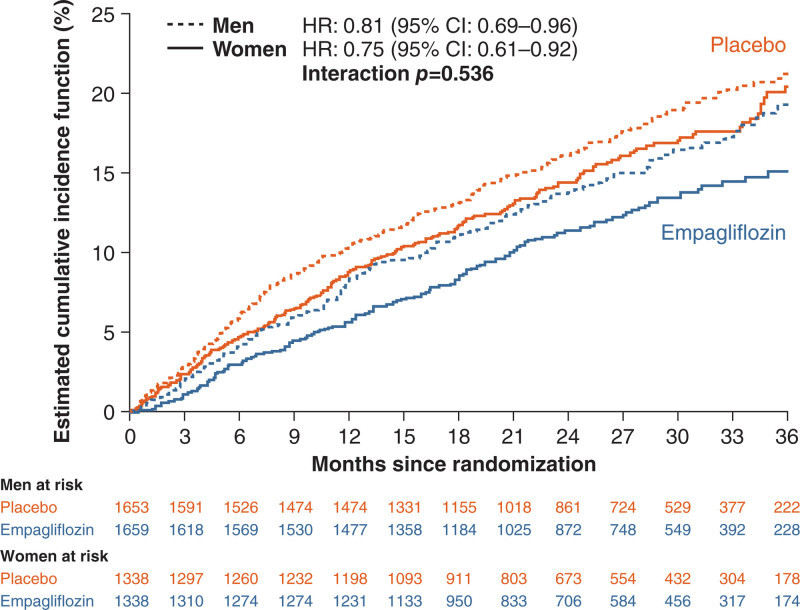
**Estimated cumulative incidence for the primary composite outcome of cardiovascular death or heart failure hospitalization according to sex and treatment.** HR indicates hazard ratio.

Empagliflozin reduced the risk of cardiovascular death or hospitalizations for HF similarly in both sexes (HR, 0.75 [95% CI, 0.61, 0.92] for women; and HR, 0.81 [95% CI, 0.69, 0.96] for men; *P*_interaction_=0.54; Figure 1). Empagliflozin had a similar effect in both sexes to reduce total HF hospitalizations (HR, 0.71 [95% CI, 0.53, 0.94] for women; and HR, 0.75 [95% CI, 0.59, 0.95] for men; *P*_interaction_=0.78) and first HF hospitalization (HR, 0.70 [95% CI, 0.54, 0.89] for women; and HR, 0.72 [95% CI, 0.58, 0.89] for men; *P*_interaction_=0.84). Empagliflozin did not reduce cardiovascular death or all-cause mortality, with no differences between the sexes (*P*_interaction_=0.673 for cardiovascular death and *P*_interaction_=0.78 for all-cause mortality; Table 2).

**Table 2.**
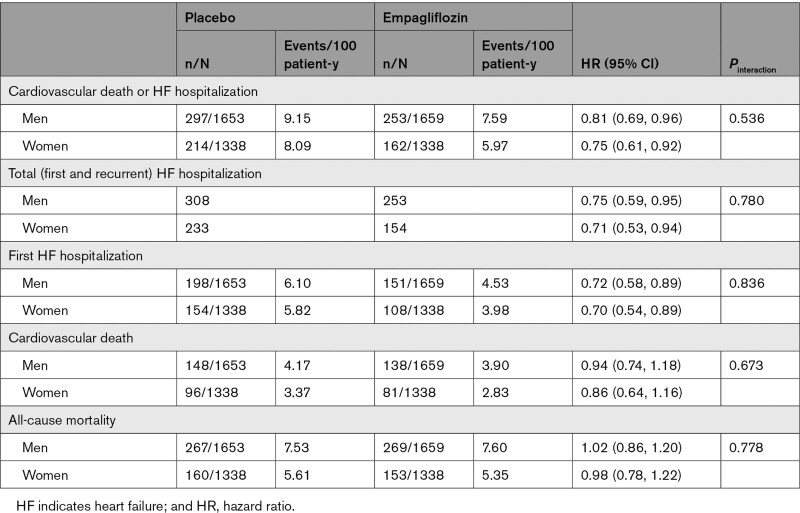
Effect of Empagliflozin on Primary and Secondary Outcomes According to Sex

**Figure 2.**
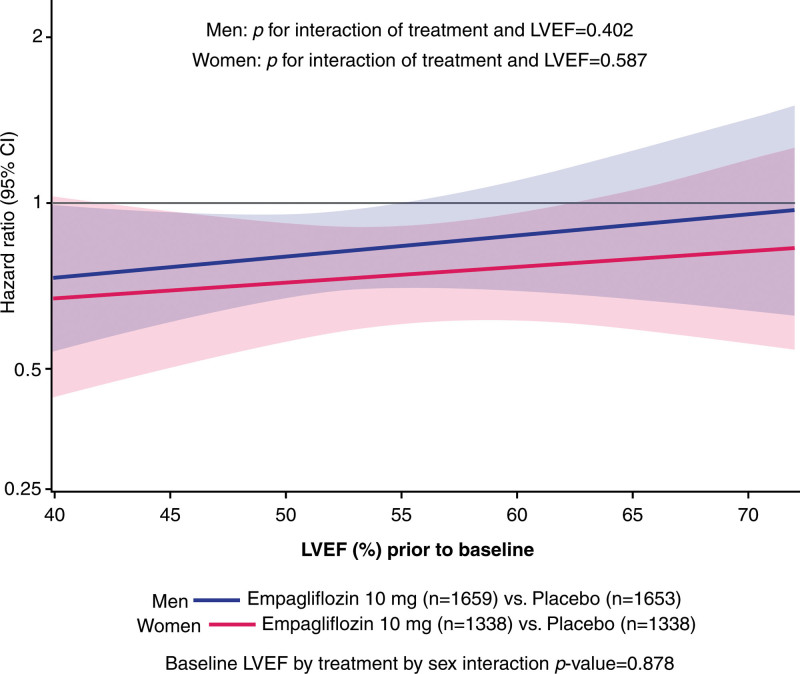
**Effect of empagliflozin on the primary composite outcome of cardiovascular death or heart failure hospitalization in women and men according to LVEF.** LVEF indicates left ventricular ejection fraction.

**Figure 3.**
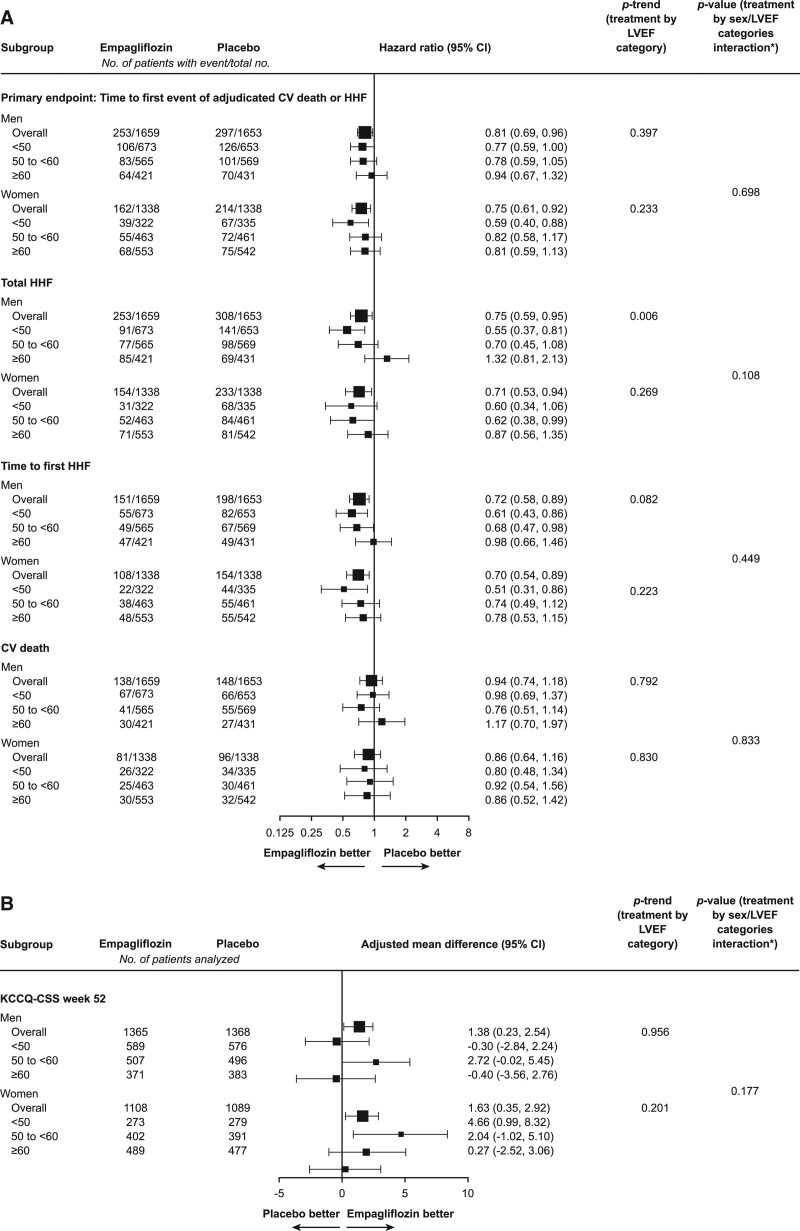
**Effect of empagliflozin on outcomes according to sex and LVEF categories.** Cardiovascular (CV; **A**) outcomes and Kansas City Cardiomyopathy Questionnaire clinical summary score (KCCQ-CSS; **B**) at 52 weeks. HHF indicates hospitalization for heart failure; and LVEF, left ventricular ejection fraction.

Treatment effect on the primary end point and total HF hospitalizations was independent of sex and LVEF categories (for treatment by subgroup of 6 categories of combination of sex and LVEF interaction for the primary end point, *P*=0.70; for total hospitalization for HF, *P*=0.108) with some tendency toward potential influence of ejection fraction on the effect of the drug on total hospitalizations for HF in men (*P*_trend_=0.006) but not in women (*P*=0.27). Figures 2 and 3 and Figures S1 through S3 provide additional details.

#### Health Status

**Figure 4.**
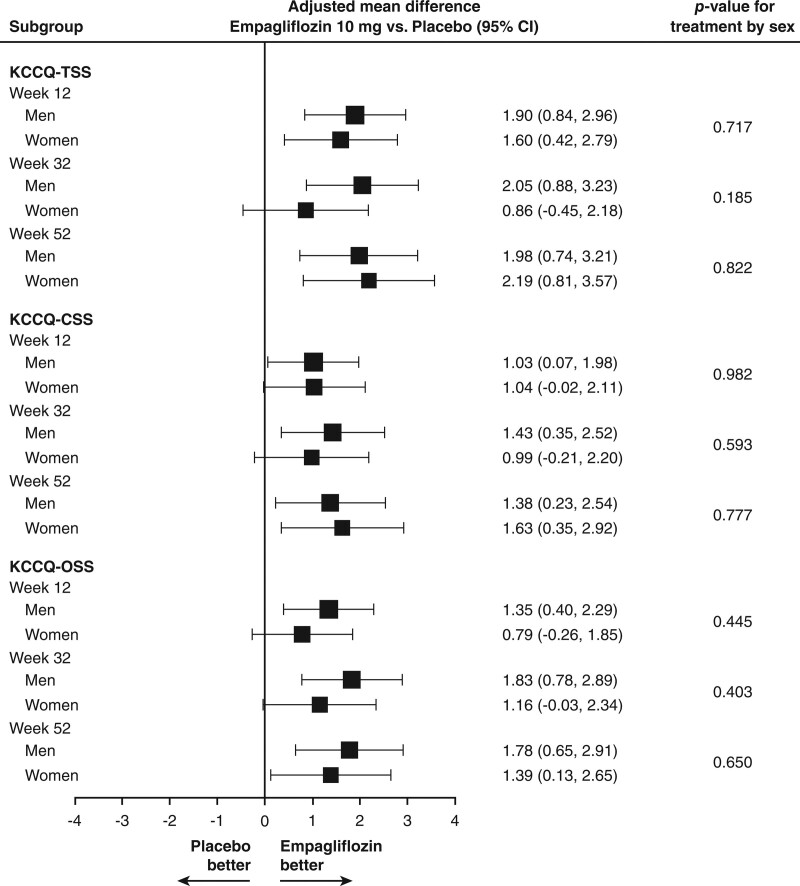
**Effect of empagliflozin on health status at 12, 32, and 52 weeks according to sex.** CSS indicates clinical summary score; KCCQ, Kansas City Cardiomyopathy Questionnaire; OSS, overall summary score; and TSS, total summary score.

Empagliflozin produced a 34% higher likelihood of being in a lower NYHA functional class at 52 weeks in women and 40% higher likelihood in men (odds ratio, 1.34 [95% CI, 1.10, 1.64] in women; and odds ratio, 1.40 [95% CI, 1.17, 1.67] in men), with no difference in the response between the sexes (*P*_interaction_=0.77). Empagliflozin improved KCCQ clinical summary score at 52 weeks to a similar degree (1.63 in women and 1.38 in men; *P*_interaction_=0.78). Similar results were seen for KCCQ overall summary score and total symptom score and at earlier time points (Figure 4).

#### Physiological Measures

The effects of empagliflozin on body weight, systolic blood pressure, NT-proBNP, uric acid, and hematocrit are shown in Table 3; no significant interactions with sex were observed.

**Table 3.**
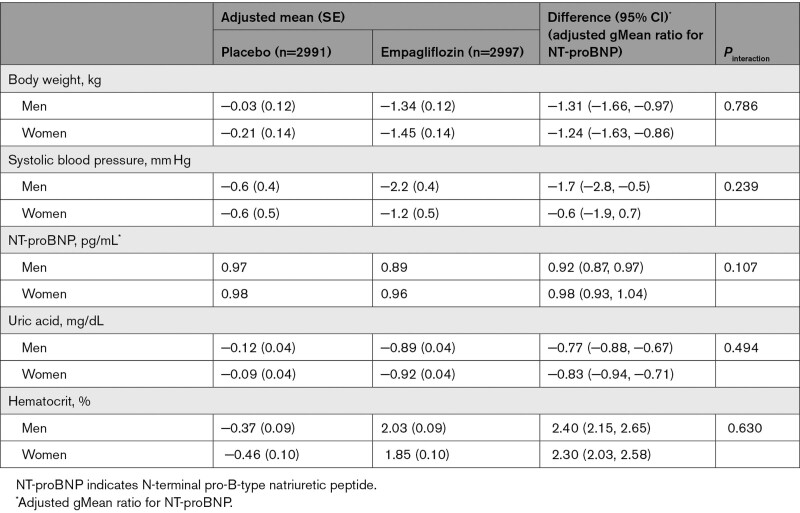
Effect of Empagliflozin on Physiological Outcomes at 52 Weeks According to Sex

#### Change in Diuretic Therapy

The effects of empagliflozin on increase in dose, decrease in dose, initiation, and time to permanent discontinuation of diuretic dose are shown in Table S2; no significant interactions with sex were observed.

#### Safety Outcomes

The effect of empagliflozin on safety outcomes according to sex is outlined in Table S3.

## Discussion

HFpEF is predominantly a disease of women, and sex influences the natural history of the disease and potentially the response to many treatments. In previous studies, women have worse health status but a lower rate of major adverse outcomes than men. These relationships were confirmed by our current analyses of the EMPEROR-Preserved trial. Differences between men and women are likely related to between-sex differences in response to hemodynamic stresses and systemic inflammation, 2 key determinants of HFpEF. Women exhibit higher pulmonary venous pressures with volume loading, possibly because they have a greater limitation of systemic venous capacitance, potentially explaining why diuretics were used more frequently in women in the EMPEROR-Preserved trial. Women show a greater degree of arterial stiffness, more impaired ventricular-vascular coupling, and more striking left ventricular concentric remodeling with pressure overload than men, and it is noteworthy that hypertension was more common (but ischemia was less common) in women than in the men in our trial. Left ventricular volumes are smaller in women than in men; thus, women are more reliant on a higher ejection fraction to maintain stroke volume and cardiac output. Indeed, ejection fractions were higher in women than in men in EMPEROR-Preserved. Furthermore, in our trial, women were more likely to be obese, and previous studies have shown that, compared with men, women are more likely to experience systemic inflammation and to show increases in proinflammatory cytokines in response to increases in body fat. Women have greater volumes of epicardial or intramyocardial fat than men and are more likely to show adverse changes in cardiac structure and function in response to systemic inflammation and metabolic disorders. These pathophysiological distinctions make it incumbent to determine whether sex influences the responses to treatments for HFpEF.

The present analysis should be distinguished from previous analyses of the EMPEROR-Preserved trial. The EMPEROR-Preserved, the largest HFpEF clinical trial to date, provided us with a unique opportunity to report the key baseline characteristics and the natural history of patients with HFpEF according to sex. Previous reports have demonstrated the benefit of empagliflozin by sex for the primary outcome of cardiovascular death and hospitalization for HF. We add to this by showing consistency of treatment effect on the primary end point regardless of sex and LVEF category and demonstrate that this benefit extends to other key prespecified clinical outcomes, health status, and physiological markers.

Previous trials have raised the possibility that sex may influence the response to treatments for HFpEF. The PARAGON-HF trial reported a highly significant treatment-by-sex interaction, with women showing a greater decrease in cardiovascular death and hospitalization for HF than men when treated with a neprilysin inhibitor (treatment-by-sex interaction, *P*=0.021).^8^ Furthermore, that trial reported a meaningful treatment–by–sex–by–ejection fraction interaction; that is, women showed a more favorable treatment effect than men up to higher values for ejection fraction (eg, ejection fractions between 55% and 60%). Yet, paradoxically, the PARAGON-HF trial showed that men responded more favorably to neprilysin inhibition than women with respect to the effect of sacubitril/valsartan on health status, as assessed by KCCQ scores (treatment-by-sex interaction, *P*=0.036). In the TOPCAT trial (Treatment of Preserved Cardiac Function Heart Failure With an Aldosterone Antagonist), among patients treated in the Americas, spironolactone reduced all-cause mortality in women, but not in men, with a significant treatment-by-sex interaction; however, sex did not influence the effect of mineralocorticoid receptor antagonism on other end points in the trial.^12^ Similarly, a recent analysis of the CHARM Program (Candesartan in Heart Failure: Assessment of Reduction in Mortality and Morbidity) showed that the benefit of treatment seemed to extend to higher LVEF in women compared with men.^13^ In contrast to these reports, trials of angiotensin-converting enzyme inhibitors and angiotensin receptor blockers in HFpEF did not show any influence of sex on the effect of treatment on adverse clinical outcomes.^14–17^

The findings in the EMPEROR-Preserved trial with respect to the influence of sex stand in contrast to the reported results of the PARAGON-HF trial. In EMPEROR-Preserved, sex did not influence the effect of empagliflozin on the primary end point of cardiovascular death or hospitalization for HF (or when the components were analyzed individually) or on health status as assessed by KCCQ scores, and we noted no significant treatment–by–sex–by–ejection fraction interaction on the primary end point. The lack of an influence of sex on the effect of empagliflozin on KCCQ scores is noteworthy because men were reported to respond more favorably than women when dapagliflozin was administered to patients with a reduced ejection fraction in the DAPA-HF trial (Dapagliflozin and Prevention of Adverse Outcomes in Heart Failure). In EMPEROR-Preserved, we previously reported an influence of ejection fraction on the effect of empagliflozin on total HF hospitalizations, with attenuation of the treatment effect in patients with ejection fractions of ≥65%.^18^ The hypothesis that empagliflozin might be less effective in elderly women with hypertension was not confirmed by this analysis, which suggests that the tendency for attenuation of response to empagliflozin on the secondary end point of total HF hospitalizations in patients with the highest ejection fraction seems to be seen primarily in men (*P*_trend_=0.006) rather than women (*P*_trend_=0.27). Because cardiac amyloidosis in HFpEF is predominantly a disease of men, these findings raise the possibility that the presence of undiagnosed cardiac amyloidosis may have influenced the response to empagliflozin in the EMPEROR-Preserved trial. Participants in the trial were not prospectively screened for amyloid heart disease.

The findings of this study should be interpreted in context of its strengths and limitations. The EMPEROR-Preserved trial was a 6000-patient trial with a large proportion of women who were treated for a median of 26 months. The influences of sex and ejection fraction were prespecified as subgroups of interest in advance of unblinding, but the analysis of treatment–by–sex–by–ejection fraction was post hoc. Measurements of physiological variables that are relevant to HFpEF (eg, cardiac volumes, visceral and myocardial adiposity, cardiac amyloid) were not performed at baseline or during follow-up.

### Conclusions

Sex did not influence the effect of empagliflozin on reducing the risk of cardiovascular death or HF hospitalization or on health status as assessed by KCCQ scores, and there was no significant treatment–by–sex–by–fraction interaction on the primary end points. These findings stand in contrast to the striking influence of sex on the response to neprilysin inhibition.

## Article Information

### Acknowledgments

Administrative support was provided by Elevate Scientific Solutions, and graphical support was provided by 7.4 Limited and supported financially by Boehringer Ingelheim.

### Sources of Funding

The EMPEROR-Preserved trial was supported by the Boehringer Ingelheim and Eli Lilly and Company Diabetes Alliance.

### Disclosures

Dr Butler reports consulting fees from Boehringer Ingelheim, Cardior, CVRx, Foundry, G3 Pharma, Imbria, Impulse Dynamics, Innolife, Janssen, LivaNova, Luitpold, Medtronic, Merck, Novartis, NovoNordisk, Relypsa, Roche, Sanofi, Sequana Medical, V-Wave Ltd, and Vifor. Dr Filippatos reports lectures and/or committee member contributions in trials sponsored by Medtronic, Vifor, Servier, Novartis, Bayer, Amgen, and Boehringer Ingelheim. Dr Siddiqi reports no conflicts. Dr Ferreira reports consulting fees from Boehringer Ingelheim during the conduct of the study. Dr Brueckmann, Dr Sumin, and T. Iwata are employees of Boehringer Ingelheim. Dr Bocchi reports consultancy fees from AstraZeneca, Boehringer Ingelheim, and Servier Affaires Medicales; grant support from Bayer, Boehringer Ingelheim, Merck, and Novartis; and congress support from Laboratorios Baldacci. Dr Böhm is supported by the Deutsche Forschungsgemeinschaft (German Research Foundation; TTR 219, project No. 322900939) and reports personal fees from Abbott, Amgen, AstraZeneca, Bayer, Boehringer Ingelheim, Cytokinetics, Medtronic, Novartis, Servier, and Vifor during the conduct of the study. Dr Chopra reports personal fees from AstraZeneca, Boehringer Ingelheim, and Novartis. Dr Giannetti reports grants and personal fees from AstraZeneca; personal fees from BMS/Pfizer Alliance, Pfizer, and Abbott; grants and personal fees from Medtronic, Novartis, and Servier; and participation in clinical trials with Novartis, Servier, Amgen, and Boehringer Ingelheim outside the submitted work. Dr Januzzi reports grant support, consulting income, and participation in clinical end point committees/data safety monitoring boards from Janssen; participation in clinical end point committees/data safety monitoring boards from Boehringer Ingelheim; grant support from Novartis, Innolife, Applied Therapeutics, and Siemens Diagnostics; and consultancy fees from Novartis, Roche Diagnostics, and Abbott Diagnostics. Dr Kaul reports personal fees from Boehringer Ingelheim during the conduct of the study and personal fees from AstraZeneca, Janssen Pharmaceuticals, Merck, Novo Nordisk, GSK, Abbott, Amarin, and Novartis outside the submitted work. Dr Piña reports personal fees from Boehringer Ingelheim. Dr Ponikowski reports personal fees from Boehringer Ingelheim, AstraZeneca, Servier, Bristol Myers Squibb, Amgen, Novartis, Merck, Pfizer, and Berlin Chemie, as well as grants and personal fees from Vifor Pharma. Dr Rauch-Kröhnert reports research support and/or consulting fees from Amgen, Merck, Sanofi, and Boehringer Ingelheim. Dr Shah has received research grants from the National Institutes of Health (R01 HL107577, R01 HL127028, R01 HL140731, and R01 HL149423), the American Heart Association (No. 16SFRN28780016), Actelion, AstraZeneca, Corvia, Novartis, and Pfizer, as well as consulting fees from Abbott, Actelion, AstraZeneca, Amgen, Axon Therapeutics, Bayer, Boehringer Ingelheim, Bristol Myers Squibb, Cardiora, CVRx, Cytokinetics, Eisai, GSK, Ionis, Ironwood, Lilly, Merck, MyoKardia, Novartis, Novo Nordisk, Pfizer, Regeneron, Sanofi, Shifamed, Tenax, and United Therapeutics. Dr Senni reports consultancy fees from Abbot, Bayer, Bayer Healthcare, Merck, Novartis, and Vifor Pharma. Dr Verma holds a Tier 1 Canada Research Chair in Cardiovascular Surgery; reports receiving research grants and honoraria from Amarin, Amgen, AstraZeneca, Bayer, Boehringer Ingelheim, Bristol Myers Squibb, Eli Lilly, HLS Therapeutics, Janssen, Novartis, Novo Nordisk, PhaseBio, and Pfizer; and reports receiving honoraria from Sanofi, Sun Pharmaceuticals, and the Toronto Knowledge Translation Working Group. He is a member of the scientific excellence committee of the EMPEROR-Reduced trial (Empagliflozin Outcome Trial in Patients with Chronic Heart Failure With Reduced Ejection Fraction) and served as a national lead investigator of the DAPA-HF and EMPEROR-Reduced trials. He is the president of the Canadian Medical and Surgical Knowledge Translation Research Group, a federally incorporated not-for-profit physician organization. Dr Zhang reports personal fees from Boehringer Ingelheim during the conduct of the study. Dr Pocock reports personal fees from Boehringer Ingelheim during the conduct of the study. Dr Zannad reports personal fees from Boehringer Ingelheim during the conduct of the study; personal fees from Janssen, Novartis, Boston Scientific, Amgen, CVRx, AstraZeneca, Vifor Fresenius, Cardior, Cereno Pharmaceutical, Applied Therapeutics, Merck, Bayer, and Cellprothera outside of the submitted work; and other support from cardiovascular clinical trialists and Cardiorenal outside of the submitted work. Dr Packer reports personal fees from Boehringer Ingelheim during the conduct of the study and personal fees from Abbvie, Actavis, Amgen, Amarin, AstraZeneca, Boehringer Ingelheim, Bristol Myers Squibb, Casana, CSL Behring, Cytokinetics, Johnson & Johnson, Lilly, Moderna, Novartis, ParatusRx, Pfizer, Relypsa, Salamandra, Synthetic Biologics, and Theravance outside the submitted work. Dr Anker has received grants from Vifor; has received personal fees from Vifor, Bayer, Boehringer Ingelheim, Novartis, Servier, Impulse Dynamics, Cardiac Dimensions, and Thermo Fisher Scientific; and has received grants and personal fees from Abbott Vascular outside the submitted work.

### Supplemental Material

Tables S1–S3

Figures S1–S3

## Supplementary Material


