# Supplementary material for: Effects of Empagliflozin in Women and Men With Heart Failure and Preserved Ejection Fraction
Source: Circulation. 2022 Sep 13;146(14):1046–55. doi: 10.1161/CIRCULATIONAHA.122.059755 (PMC9528945; doi:10.1161/CIRCULATIONAHA.122.059755)
Supplement: Supplementary file 1 [file cir-146-1046-s001.pdf]

Effects of Empagliflozin in Women and Men with Heart Failure and Preserved Ejection Fraction

Javed Butler, MD, MPH, MBA; Gerasimos Filippatos, MD; Tariq Jamal Siddiqi, MBBS; João Pedro Ferreira, MD, PhD; Martina Brueckmann, MD; Edimar Bocchi, MD, PhD; Michael Böhm, MD; Vijay K. Chopra, MD; Nadia Giannetti, MD; Tomoko Iwata, MS; James L Januzzi, MD; Sanjay Kaul, MD; Ileana L. Piña, MD, MPH; Piotr Ponikowski, MD, PhD; Ursula Rauch-Kröhnert, MD; Sanjiv J. Shah, MD; Michele Senni, MD; Mikhail Sumin, MD, PhD; Subodh Verma, MD, PhD; Jian Zhang, MD, PhD, FACC, FESC; Stuart J. Pocock, PhD; Faiez Zannad, MD, PhD; Milton Packer, MD; Stefan D. Anker MD, PhD

SUPPLEMENTAL MATERIAL

SUPPLEMENTAL TABLES

Table S1. Baseline Characteristics of Patients According to Sex and LVEF Category

	41–49%			50–59%			≥60%		
	Men (n=1326)	Women (n=657)	*P* value	Men (n=1134)	Women (n=924)	*P* value	Men (n=852)	Women (n=1095)	*P* value
Age, years	69.5 (9.6)	71.2 (9.8)	<0.001	71.6 (9.3)	73.1 (9.2)	<0.001	72.6 (8.8)	73.9 (9.1)	0.001
Race			<0.001			<0.001			0.002
Asian	192 (14.5)	50 (7.6)		159 (14.0)	93 (10.1)		171 (20.1)	159 (14.5)	
Black or African American	62 (4.7)	37 (5.6)		30 (2.6)	46 (5.0)		29 (3.4)	54 (4.9)	
White	1001 (75.5)	500 (76.1)		882 (77.8)	714 (77.3)		621 (72.9)	824 (75.3)	
Other including mixed race	71 (5.4)	70 (10.7)		61 (5.4)	71 (7.7)		31 (3.6)	58 (5.3)	
Missing				2 (0.2)	0.0				
Geographic region			<0.001			0.148			0.003
Asia Pacific	154 (11.6)	33 (5.0)		131 (11.6)	77 (8.3)		154 (18.1)	137 (12.5)	
Europe	584 (44.0)	325 (49.5)		548 (48.3)	462 (50.0)		334 (39.2)	436 (39.8)	
North America	120 (9.0)	41 (6.2)		149 (13.1)	114 (12.3)		134 (15.7)	161 (14.7)	
Latin America	381 (28.7)	218 (33.2)		234 (20.6)	206 (22.3)		184 (21.6)	292 (26.7)	
Other	87 (6.6)	40 (6.1)		72 (6.3)	65 (7.0)		46 (5.4)	69 (6.3)	
KCCQ-CSS	75.8 (19.7)	65.6 (21.2)	<0.001	74.1 (19.8)	64.3 (21.5)	<0.001	74.3 (20.2)	65.3 (21.4)	<0.001
KCCQ-OSS	73.5 (20.0)	64.3 (21.1)	<0.001	72.3 (19.8)	63.3 (21.4)	<0.001	72.7 (20.1)	64.6 (21.8)	<0.001
KCCQ-TSS	78.4 (20.1)	69.9 (22.3)	<0.001	76.7 (21.0)	68.4 (23.0)	<0.001	76.4 (20.8)	68.3 (22.7)	<0.001
HF hospitalization within 1 year	334 (25.2)	153 (23.3)	0.355	284 (25.0)	209 (22.6)	0.200	183 (21.5)	206 (18.8)	0.144
BMI, kg/m^2	28.9 (5.3)	30.0 (6.1)	<0.001	29.9 (5.7)	30.7 (6.1)	0.001	29.6 (5.7)	30.3 (6.3)	0.005
Ejection fraction at screening, %	44.7 (2.3)	45.1 (2.3)	0.002	53.6 (2.9)	54.0 (2.9)	0.003	63.9 (4.4)	65.1 (5.3)	<0.001
New York Heart Association Class II	1122 (84.6)	506 (77.0)	<0.001	957 (84.4)	721 (78.0)	0.001	729 (85.6)	848 (77.4)	<0.001

Systolic blood pressure, mm Hg	130.5 (15.2)	130.8 (15.2)	0.636	132.7 (15.5)	132.3 (16.0)	0.555	132.1 (15.6)	132.7 (16.2)	0.356
Diastolic blood pressure, mmHg	76.4 (10.3)	76.5 (10.2)	0.785	76.1 (10.5)	75.3 (10.9)	0.095	75.2 (10.7)	75.0 (10.6)	0.577
Heart rate, bpm	70.0 (11.5)	72.4 (12.5)	<0.001	69.9 (11.7)	71.1 (12.0)	0.021	69.7 (11.8)	70.1 (11.9)	0.451
Hypertension	1156 (87.2)	600 (91.3)	0.006	1028 (90.7)	847 (91.7)	0.421	778 (91.3)	1015 (92.7)	0.263
Diabetes mellitus	682 (51.4)	343 (52.2)	0.745	570 (50.3)	437 (47.3)	0.180	430 (50.5)	476 (43.5)	0.002
Atrial fibrillation	576 (43.4)	310 (47.2)	0.111	639 (56.3)	502 (54.3)	0.391	471 (55.3)	559 (51.1)	0.062
Coronary artery disease	633 (47.7)	198 (30.1)	<0.001	479 (42.2)	239 (25.9)	<0.001	315 (37.0)	230 (21.0)	<0.001
Ischemic etiology	715 (53.9)	268 (40.8)	<0.001	442 (39.0)	258 (27.9)	<0.001	254 (29.8)	180 (16.4)	<0.001
ACE-I, ARB, ARNI	1116 (84.2)	569 (86.6)	0.152	914 (80.6)	745 (80.6)	0.987	638 (74.9)	850 (77.6)	0.157
Diuretic*	1140 (86.0)	592 (90.1)	0.009	950 (83.8)	837 (90.6)	<0.001	698 (81.9)	946 (86.4)	0.007
Beta-blocker	1194 (90.0)	598 (91.0)	0.489	975 (86.0)	796 (86.1)	0.913	713 (83.7)	891 (81.4)	0.183
Mineralocorticoid receptor antagonist	595 (44.9)	329 (50.1)	0.029	395 (34.8)	355 (38.4)	0.093	241 (28.3)	329 (30.0)	0.397
Statin	993 (74.9)	450 (68.5)	0.003	817 (72.0)	596 (64.5)	<0.001	594 (69.7)	681 (62.2)	0.005
Hemoglobin, g/dL	13.9 (1.6)	13.0 (1.4)	<0.001	13.7 (1.6)	12.8 (1.4)	<0.001	13.5 (1.6)	12.8 (1.4)	<0.001
eGFR, ml/min/1.73 m^2	64.7 (20.3)	59.7 (19.9)	<0.001	61.7 (19.6)	57.7 (19.2)	<0.001	60.6 (19.0)	57.7 (19.7)	0.001
NT-proBNP (pg/mL)	984 (530–1825)	1112 (609–1934)	0.146	1046 (530–1772)	954 (481–1721)	0.046	908 (483–1663)	856 (431–1591)	0.249

Data are mean (SD) or number (%) except that NT-proBNP is median (IQR). Race was self-reported. Those who identified with more than one race or with no race were classified as 'other'. Angiotensin receptor blocker is excluding valsartan when taken with sacubitril because sacubitril/valsartan is shown as angiotensin receptor neprilysin inhibitor. KCCQ-CSS indicates Kansas City Cardiomyopathy Questionnaire-clinical summary score; ACE-I, angiotensin-converting enzyme inhibitor; ARB, angiotensin receptor blocker; BMI, body mass index; CAD, coronary artery disease; eGFR, estimated glomerular filtration rate; HF, heart failure; KCCQ-OSS, Kansas City Cardiomyopathy Questionnaire-overall summary score; KCCQ-TSS,

Kansas City Cardiomyopathy Questionnaire-total summary score; NT-proBNP, N-terminal pro-B-type natriuretic peptide.

* Excluding mineralocorticoid receptor antagonists.

Table S2. Effect of Empagliflozin on Change in Diuretic Therapy Over Time According to Sex.

	**Placebo**		**Empagliflozin**		**HR (95% CI)**	***P* interaction**
	**n/N**	**Events/100 patient-yr**	**n/N**	**Events/100 patient-yr**		
Increase in dose[a]						
Men	280/1285	11.94	213/1300	8.52	0.70 (0.59, 0.84)	0.417
Women	247/1117	12.19	196/1107	9.47	0.78 (0.65, 0.94)	
Decrease in dose[a]						
Men	169/1285	6.84	217/1300	8.92	1.28 (1.05, 1.57)	0.503
Women	155/1117	7.23	172/1107	8.31	1.16 (0.93, 1.44)	
Initiation[b]						
Men	113/368	18.40	92/359	14.41	0.79 (0.60, 1.04)	0.418
Women	82/221	23.35	61/231	15.66	0.66 (0.47, 0.92)	
permanent discontinuation[a]						
Men	78/1285	2.99	122/1300	4.68	1.56 (1.18, 2.08)	0.464
Women	58/1117	2.53	77/1107	3.43	1.32 (0.94, 1.86)	

[a] Only patients with diuretics use at baseline

[b] Only patients without diuretics use at baseline

Table S3. Safety Outcomes According to Sex

	Men, N (%)		Women, N (%)	
	Empagliflozin (n=1658)	Placebo (n=1653)	Empagliflozin (n=1338)	Placebo (n=1336)
Patients with any adverse event	1412(85.2)	1412 (85.4)	1162 (86.8)	1173 (87.8)
Patients with any serious adverse event	826 (49.8)	885 (53.3)	610 (45.6)	658 (49.3)
Hypotension	168 (10.1)	149 (9.0)	143 (10.7)	108 (8.1)
Symptomatic hypotension[a]	108 (6.5)	90 (5.4)	89 (6.7)	66 (4.9)
Acute renal failure	198 (11.9)	198 (12.0)	165 (12.3)	186 (13.9)
Ketoacidosis[b]	3 (0.2)	2 (0.1)	1 (0.1)	3 (0.2)
Hepatic injury	61 (3.7)	86 (5.2)	54 (4.0)	69 (5.2)
Hypoglycemic events[c]	41 (2.5)	41 (2.5)	32 (2.4)	37 (2.8)
In patients with diabetes mellitus	35 (4.1)	34 (4.1)	28 (4.6)	32 (5.0)
In patients without diabetes mellitus	6 (0.7)	7 (0.8)	4 (0.5)	5 (0.7)
Urinary tract infections	99 (6.0)	79 (4.8)	198 (14.8)	164 (12.3)
Complicated urinary tract infections	17 (1.0)	26 (1.6)	40 (3.0)	19 (1.4)
Genital infections	32 (1.9)	9 (0.5)	35 (2.6)	13 (1.0)
Complicated genital infections	8 (0.5)	7 (0.4)	0 (0.0)	1 (0.1)
Bone fractures	52 (3.1)	49 (3.0)	82 (6.1)	77 (5.8)
Events leading to lower limb amputation[a]	14 (0.8)	17 (1.0)	2 (0.1)	6 (0.4)

Shown are adverse events up to 7 days following discontinuation of study medication, but lower limb amputations were shown up to the end of the trial.

[a] Investigator-defined events

[b] All events occurred in patients with diabetes mellitus at baseline

[c] Hypoglycemic AEs with a plasma glucose value of ≤70 mg/dL or that required assistance

SUPPLEMENTAL FIGURES

Figure S1. Spline regression model across LVEF according to sex for first hospitalization for heart failure or CV death.

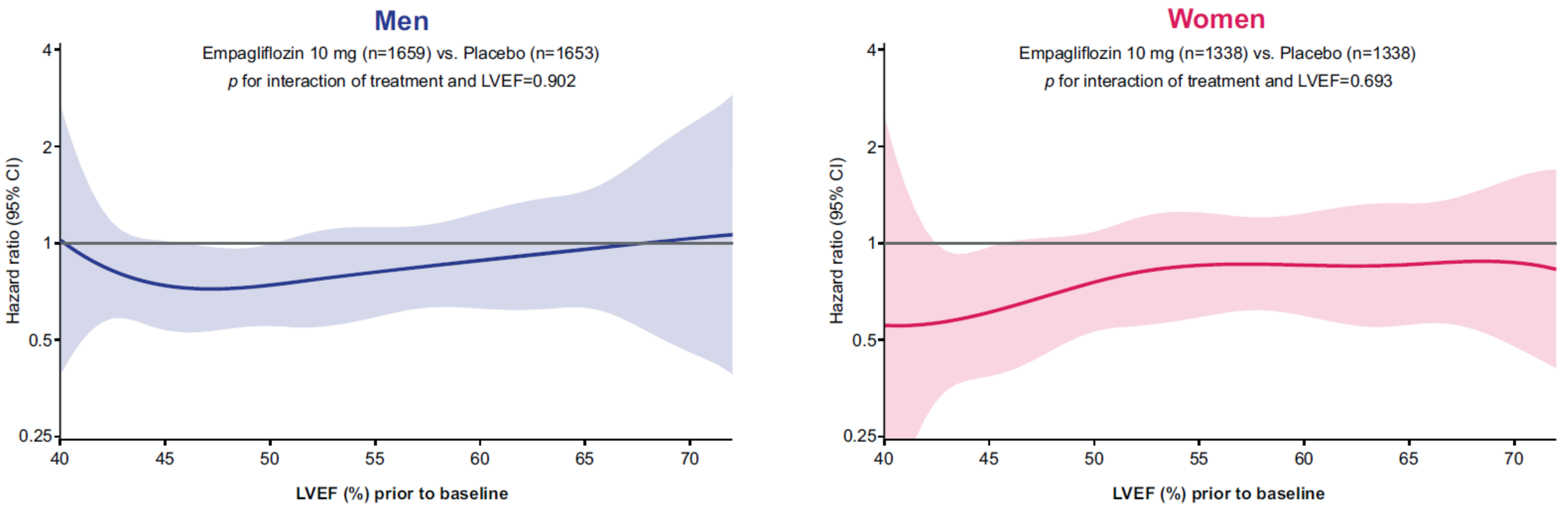

Baseline LVEF by treatment by sex interaction *p*-value=0.823

CI, confidence interval; LVEF, left ventricular ejection fraction, CV, cardiovascular.

Figure S2. Spline regression model across LVEF according to sex for first hospitalization for heart failure.

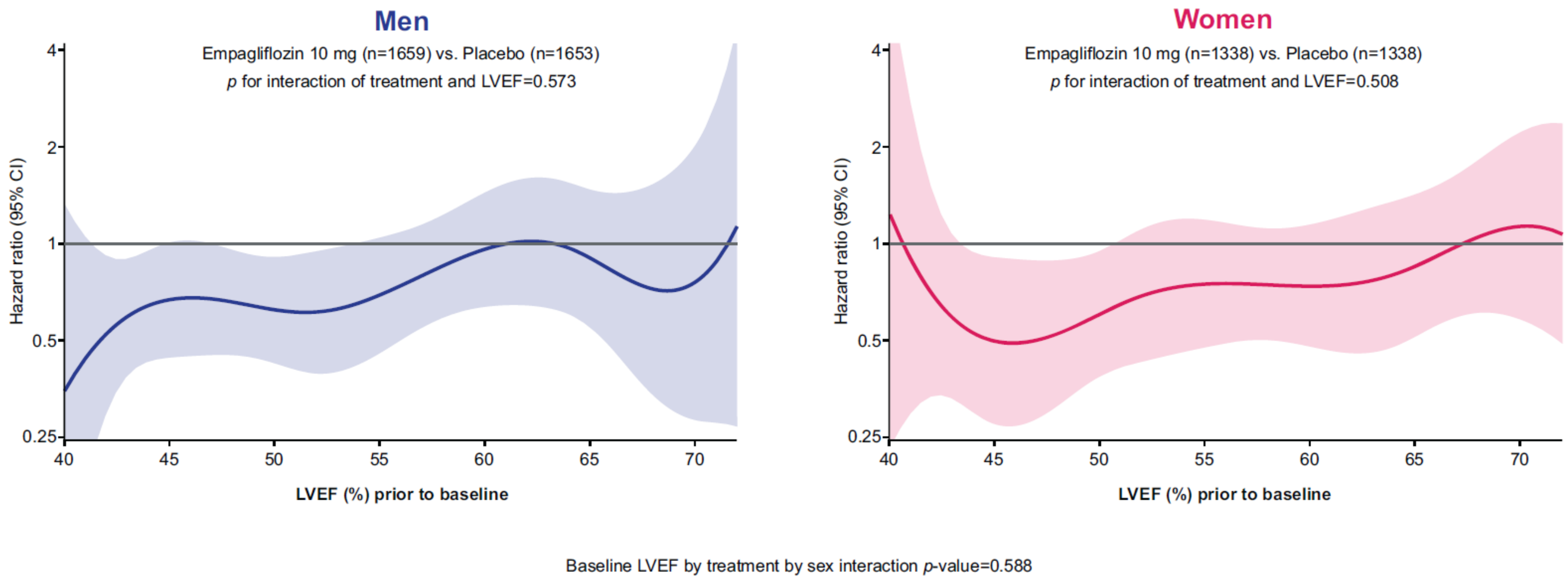

CI, confidence interval; LVEF, left ventricular ejection fraction.

Figure S3. Spline regression model across LVEF according to sex for first and recurrent hospitalization for heart failure.

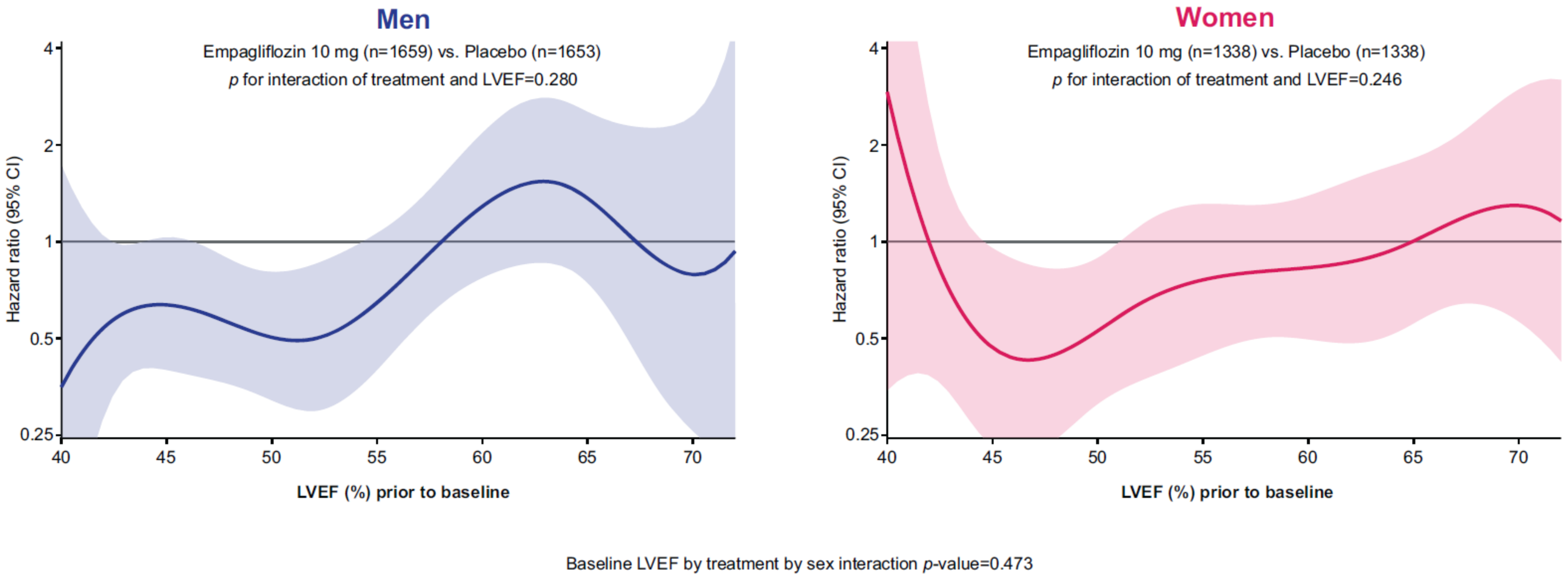

CI, confidence interval; LVEF, left ventricular ejection fraction.
